# The Need for Formal Evidence Synthesis in Food Policy: A Case Study of Willingness-to-Pay

**DOI:** 10.3390/ani7030023

**Published:** 2017-03-10

**Authors:** Beth Clark, Lynn J. Frewer, Luca A. Panzone, Gavin B. Stewart

**Affiliations:** School of Agriculture Food and Rural Development, Newcastle University, Newcastle upon Tyne, NE1 7RU, UK; b.clark@newcastle.ac.uk (B.C.); lynn.frewer@newcastle.ac.uk (L.J.F.); luca.panzone@newcastle.ac.uk (L.A.P.)

**Keywords:** farm animal welfare, meta-analysis, willingness-to-pay, publication bias

## Abstract

**Simple Summary:**

Meta-analysis is a statistical technique used to combine results of different studies on the same topic. These methods are gaining increasing popularity in the field of consumer behaviour; however, the issue of publication bias, which is the tendency for the study outcome to influence whether the research is published or not, has received little attention in this field. This research, therefore, looked to explore publication bias in willingness-to-pay, using the example of willingness-to-pay for farm animal welfare. A systematic search of four online databases led to the inclusion of 54 studies for analysis. Publication bias was assessed by using four different tests. The results indicate that publication bias is present in the literature, with willingness-to-pay for farm animal welfare being overestimated as a result. Given the use of willingness-to-pay in policy, stakeholders should ensure that results of meta-analysis are assessed for publication bias.

**Abstract:**

Meta-analysis is increasingly utilised in the understanding of consumer behaviour, including in relation to farm animal welfare. However, the issue of publication bias has received little attention. As willingness-to-pay (WTP) is widely used in policy, it is important to explore publication bias. This research aimed to evaluate publication bias in WTP, specifically public WTP for farm animal welfare. A systematic review of four databases yielded 54 studies for random effects meta-analysis. Publication bias was assessed by the Egger test, rank test, contour-enhanced funnel plots, and the Vevea and Hedges weight-function model. Results consistently indicated the presence of publication bias, highlighting an overestimation of WTP for farm animal welfare. Stakeholders should be wary of WTP estimates that have not been critically evaluated for publication bias.

## 1. Introduction

Evidence synthesis methods, such as systematic reviews and meta-analyses, are being more frequently adopted in the study of consumer behaviour for a number of reasons. These include increases in the volume of information, the increased focus on research which delivers the basis for evidence-based policy and practice, and the need to assess and present the totality of evidence in relation to a given topic to all stakeholders concerned [1,2,3]. This synthesis of results is especially important as most individual studies are not large enough, or are too contextual, to identify trends or enable generalisations to be made [4,5], particularly in the context of applied social science research. Additionally meta-analysis provides increased power for analysis, a formal means of exploring heterogeneity, critical appraisal to inform future research and formalised sensitivity analysis, whereby sensitivity analysis is a repeat of the meta-analysis using an alternative set of studies or processes for aspects that were deemed unclear, so as to provide an indication of the robustness of the results. e.g., for studies whose eligibility was unclear [6,7]. These methods have had transformative effects in other disciplines, such as medicine, social reform, and education, yet systematic review and meta-analysis in topics related to food policy remain insufficient and are normally only done in part.

In addition to providing robust and transparent forms of evidence synthesis, meta-analysis has the added advantage of identifying additional weaknesses in the evidence base, such as publication bias. This has, in the social sciences, been defined as “*the tendency toward preparation, submission and publication of research findings based on the nature and direction of the research results*” [8], and is when the publically-available literature is not fully representative of all of the completed studies of a given topic of interest [9]. Failure to publish results could occur for a number of reasons [10,11]. These include researchers failing to write up non-significant or negative results (i.e., the “file drawer” problem) [12], or such results being peer reviewed less favourably with editors being less likely to publish results which do not support existing findings [6]. Conversely, studies with statistically significant and supportive outcomes are more likely to be published [13], with the same piece of work likely to be published on multiple occasions [14]. This failure to obtain a true representative sample of all those addressing a specific topic can threaten the validity of the meta-analysis [15,16,17], and can lead to the formation of unreliable conclusions [18,19], including a distorting effect on cumulative knowledge and evidence-based practice. It is important to note that this is not a failure of the search process to locate a representative sample of studies [20], but rather a problem with the availability of the literature in the first instance due to non-publication.

Publication bias is a widely acknowledged problem [21]. Despite the majority of evidence coming from the health sciences [20], there is also evidence of a growing body of evidence in the social science literature [22], for example, in economics [17,19] and psychology [23]. However, publication bias is rarely tested for in meta-analyses within economics [24,25], nor the wider consumer behaviour literature [26,27,28]. Where it has been explored, recommended tests [6] have often not been used [29], and publication bias has been missed when tested [30]. It is important to conduct a formal assessment of publication bias so as to provide an indication as to the robustness of the results [9] and the strength of evidence of the conclusions made. 

Willingness-to-pay (WTP) is defined as the maximum price an individual is willing to sacrifice to obtain a certain benefit or to avoid an issue, event, or loss [31], and can be taken as a measure of value of goods or services to an individual [32]. It is especially useful for evaluating non-market goods [33] and is a widely used tool as part of cost-benefit analysis in policy-making. However, a number of criticisms of WTP exist. These include the assumption that individuals are the best judge of their own wellbeing [33], that there is no common unit, scale, or monetary values to assess the value of ethical issues and values associated with welfare or the environment [34], and that WTP estimates are influenced by the wealth of the individual providing them. A number of types of bias exist in WTP studies, including hypothetical bias [33], non-response bias [35], and information bias [33]. There are also the advantages and disadvantages of the individual methods, themselves, which are partially attributed to different types of bias [36].

The majority of these criticisms of the WTP method are well known [33] and are commonly taken into consideration. However, publication bias still continues to receive relatively little attention. This paper, therefore, aims to explore publication bias in the WTP literature. This will be conducted using the example of WTP for improved farm animal welfare (FAW).

## 2. Methods 

**Figure 1:**
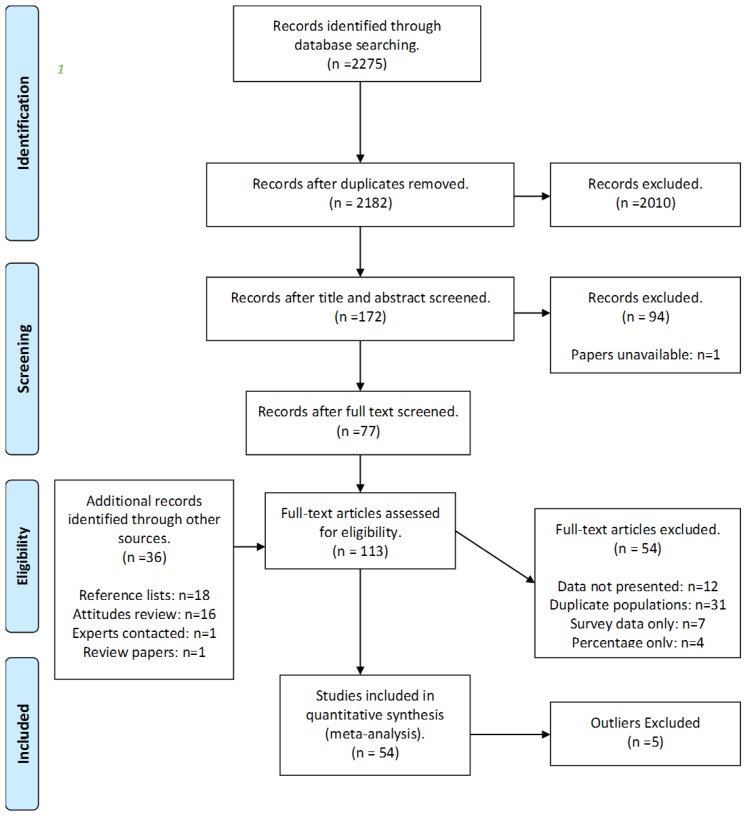
A PRISMA flow diagram of the search process (a separate review of public attitudes towards farm animal welfare was conducted simultaneously to this, and relevant references identified through this search process were included in this review. The review paper in question was [39]).

In order to provide transparency and enable feedback, a protocol for the systematic review was published online prior to commencing the literature search. Four databases were searched using tailored strings of keywords (Supplementary Table S1); Scopus, ISI Web of Knowledge, AgEcon Search, and Google Scholar, the latter of which facilitated the identification of “grey” literature (grey literature is literature that is unpublished or not published in traditional academic journals. Examples include government reports or PhD thesis). In addition, reference lists of studies identified through these search approaches were checked, and key authors in the field were contacted to identify any additional studies not returned from the original search process, including unpublished research. All returned studies were exported to Endnote (Thomson Reuters, New York, NY, USA ) [37] for further analysis. The face validity of each of the searches run were checked by examining the search results for papers included in a previous review [29], and this helped to establish the most appropriate strings of search words to use. The identified studies were screened at the title and abstract and at full text level (i.e., a two-stage process), using a set of pre-determined inclusion criteria based on the PICO (population, intervention, control, outcome) model to establish their relevance to the research questions. This led to the inclusion of 54 studies (Supplementary Information S1). An overview of the search process can be found in the PRISMA flow diagram in Figure 1 [38], including the number of studies included and excluded at each stage.

Only quantitative empirical studies were included in the review, specifically those that examined the public’s WTP for FAW. This was deemed to be any monetary measure for any animal-based product or related tax, in relation to an improvement of any measure of welfare, which respondents could perceive as improving the animals’ lives. All animal types and only studies written in English were included. A measure of FAW was deemed to be anything described to participants as altering the welfare of farm animals in some way, usually for the better, ranging from specific measures, such as stocking density, e.g., free-range, to vague descriptors, such as general improvements to overall welfare. Most studies reported multiple measures of WTP and all were extracted for analysis. A broad range of methods used to measure WTP were considered for inclusion in the review. This included, but was not limited to, revealed preference measures (market data, experimental auctions) and stated preference measures (conjoint analysis, contingent valuation studies, choice experiments). Studies with duplicate populations (where the same data was presented in two or more publications) were removed, with the study with the lowest critical appraisal score or which reported the fewest WTP or socio-demographic measures being excluded from the current analysis. The critical appraisal score was based on the assessment of a number of quality criteria that had the potential to impact on the results of the study; the WTP method used, the economic model used, the sample population and the sampling technique for each study. No studies were excluded based on the critical appraisal, with the findings being taken into account during the evidence synthesis when assessing the overall strength of evidence as part of the GRADE (Grading of Recommendations, Assessment, Development and Evaluation) analysis [40].

WTP values were extracted from each study as the price premium paid for the specified product, and adjusted values for inflation using the consumer price index. Values were then converted into a standardised currency before calculating effect sizes for each premium to provide a common currency unit. Finally the unit for analysis was standardised by using effect sizes, calculated as the mean WTP divided by the standard deviation for each WTP measure [41]. When the standard deviation was not available, the standard error and 95% CI were used to calculate this, as per Lipsey and Wilson [7], and these are referred to as complete case values. Where standard deviations, standard errors, or 95% CI were not reported we were able to impute the variance of the data using R (referred to as imputed values) [42]. Five outliers (any paper with an effect size more than 10 times the pooled effect size) were identified and removed from the dataset before continuing with the analysis. The meta-analysis was conducted using the open source ‘metafor’ package [42] in ‘R’ (R Core Team, Vienna, Austria, 2015). A random-effects meta-analysis was used to calculate the effect size [43], being considered more robust when heterogeneity is high. The random effects model is a special case of the general linear model, and provides an unconditional inference about a larger set of studies, for which the sample of studies in the meta-analysis is only a random sample of the totality of evidence [43]. All analyses were conducted using the restricted maximum likelihood estimator, which is better suited for smaller sample sizes [42]. 

A cumulative meta-analysis was also conducted on the aggregated effect size for each study (Supplementary Figure S1). A positive effect size indicates a WTP a price premium for products produced to above minimum standards of welfare. Forest plots were generated for each analysis to establish the extent of any inconsistency in the data [44], in relation to the size of study effects. 

There are three main mays of dealing with publication bias [45]; (1) to use methods that prevent publication bias from occurring; (2) methods that attempt to detect the presence of publication bias and; (3) attempts to compensate for publication bias, by establishing what the combined effect sizes would be if the bias (or censorship) had not been present. As there are very few measures in social sciences to prevent publication bias from occurring (such as registries, and mandatory protocols), efforts were focused on the latter two ways of addressing publication bias. As none of the methods for detecting publication bias are thought to be entirely satisfactory [4,20], four different methods were used in the analysis in order to provide a triangulation of results, whereby the use of the four methods provides a means of validation data through the cross-checking of the results. All tests were performed on the aggregated, complete case, and overall study values (both complete case and imputed values) to enable a comparison. All tests were conducted with the null hypothesis that there is no asymmetry in the data, as the finding of asymmetry would indicate publication bias in the data, as described during the description of funnel plots. Results were also conducted on different sub-groups (animal type, region, population, and methodological variables) except for the comparison of the effect size between published and grey literature due to small sample sizes, and these are displayed in Supplementary Table S2.

First contour enhanced funnel plots were used to establish whether publication bias was present. Funnel plots aid in the interpretation of results and are the most common means of identifying publication bias. These are scatter graphs which plot the study’s effect size typically against the study’s size [15] or some measure related to the study size, such as standard error, with the estimate precision increasing as the sample size increases [14], as the study result is less likely to have occurred by chance [46]. If no publication bias is present, the dispersion of studies should be shaped like a funnel, with the typically more variable and numerous smaller studies on the bottom, and the larger studies being nearer the top. If publication bias is present asymmetry around the average effect size (and so the funnel) occurs, with studies with small or non-significant results (usually in the bottom left-hand corner) typically missing [13]. However, it should be noted that the detection of asymmetry or a significant results is not necessarily to do with bias and should be considered as an exploration of small study effects [6] from a variety of sources [47,48]. Contour-enhanced funnel plots aid with this [14], examining the context of the statistical significance of the results, looking at the distribution of the studies to see whether the areas of missing studies are due to statistical significance [44].

Second, the Egger test [47] was used to check for publication bias. The Egger test uses a regression-based approach to test for asymmetry in the data by plotting the included effect sizes against a measure of their variance, and examines whether the intercept of the regression line significantly deviates from zero. When publication bias is present the regression line will not run through the origin, leading to a significant result and indicating publication bias in the data. 

Thirdly, the Begg and Mazumder rank test [49] was used. This uses the Kendall’s rank correlation to examine the relationship between the effect sizes and their sampling variances. A significant correlation is taken as indicating that publication bias is present in the data. 

The Vevea and Hedges model [45] was also used to establish the difference between adjusted and unadjusted effect sizes. This weight function model corrects for publication bias by using a weight function to represent the process of selection. The model has two parts that incorporate a model for the distribution of the effect size estimates before selection occurs and a model for the selection process which will describe how it has affected the distribution of the estimates [45]. These models are quite complex and involve a lot of computation and are, therefore, less widely used than the previously-mentioned tests of publication bias. However, the Shiny app by Coburn and Vevea [50], provides a more accessible means of utilizing the Vevea and Hedges model. *p* value cutpoints of 0.05 were used in the analysis due to this being a commonly used and widely understood value, with the weights used for analysis being the default selection of the Shiny application, in which the first weight fixed to 1, and all subsequent weights are interpreted relative to this [50]. Hedges and Vevea [15] also recommend examining the effect size of published versus unpublished studies (i.e., peer reviewed vs. non-peer reviewed) to see if there are any differences in effect size, and this was the final test for publication bias.

## 3. Results

The 54 studies included in the final analysis provided 335 measures of WTP, with studies on average providing six WTP measures. Seventeen of these studies (31.5%) failed to provide variances for their WTP estimates, leading to their measures being imputed. These will be referred to as imputed values, with studies that did provide variances referred to as complete case values. Average effect sizes for each study were calculated and these will be referred to as aggregated values. The majority of studies (*n* = 47) were peer reviewed journal articles, with the remaining seven being a mixture of reports, theses, and conference papers. The results for all tests of publication bias are presented for the complete case (*n* = 227), overall (*n* = 335, complete case and imputed values), and aggregated values (*n* = 54), respectively. The average effect sizes were calculated for each analysis as the standardised mean difference (mean WTP divided by the corresponding variance) and are as follows: complete case analysis (I^2^ 99.71%, effect size 0.6302 (95% CI 0.5016, 0.7587), significance *p* < 0.0001), overall data analysis (99.76%, 0.5709 (0.4599, 0.6819), *p* < 0.0001), and aggregated value analysis (99.72%, 0.6135 (0.4106, 0.8524), *p* < 0.0001). Effect sizes for each analysis were reasonably consistent with one another and all indicated that the public are WTP a small price premium for products produced with higher than minimum animal welfare standards, e.g., free-range eggs, compared to barn eggs. Full analysis and discussion of the results can be obtained by contacting the corresponding author. Results from the assessments of publication bias are presented below and are summarised in Table 1 and Table 2. 

**Figure 2:**
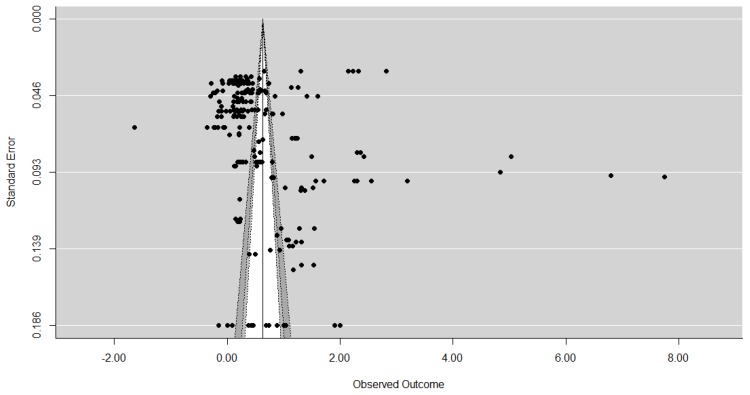
A contour enhanced funnel plot for complete case values (*n* = 227). Light grey is <1% significance, medium grey is 1%–<5% significance, dark grey is 5%–10% significance, and the white is the area of non-significance.

**Figure 3:**
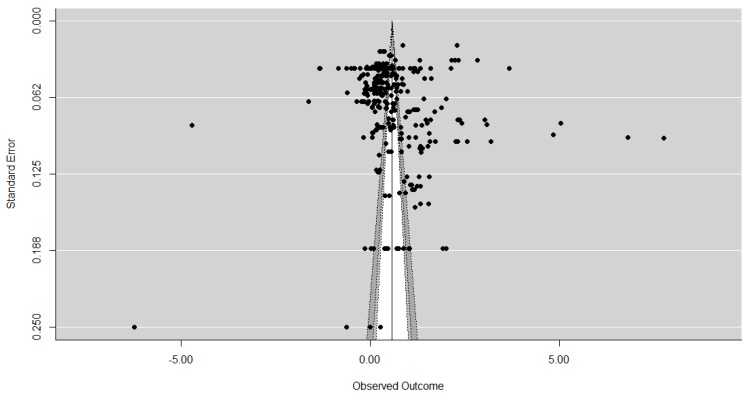
A contour enhanced funnel plot for overall values (*n* = 335). Light grey is <1% significance, medium grey is 1%–<5% significance, dark grey is 5%–10% significance, and the white is the area of non-significance.

**Figure 4:**
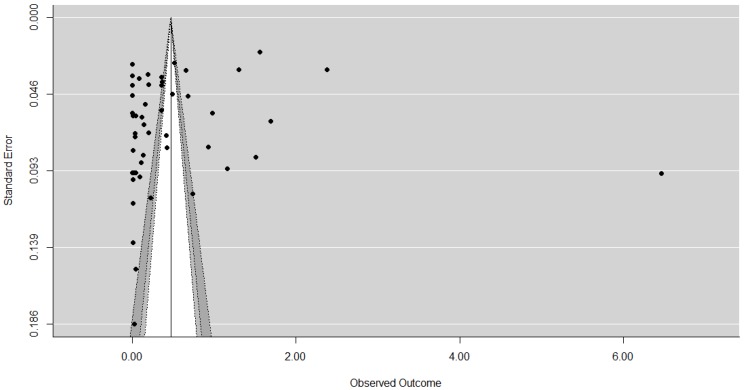
A contour enhanced funnel plot for aggregated values (*n* = 54). Light grey is <1% significance, medium grey is 1%–<5% significance, dark grey is 5%–10% significance, and the white is the area of non-significance.

The contour-enhanced funnel plots for each sample are displayed in Figure 2, Figure 3 and Figure 4 and, as expected, the larger studies show less variation in their effect size estimates compared the smaller studies. The results of the contour-enhanced funnel plots for the complete case, overall, and aggregated values demonstrate asymmetry indicating that publication bias is present in the results. The contours on the plots indicate that asymmetry occurs on the left hand side of the plot, indicating studies with smaller than average effect sizes are missing. These missing studies on both sides appear to be in the area of significance at the 5% level and below to the left of the average effect size. In addition, there appear to be few small studies with non-significant results, as indicated by the lack of studies in the white region in the centre of the funnels.

The results of the Egger’s test are significant for the complete case values only (z = 3.7300, *p* = 0.0002), leading to rejection of our null hypothesis (H_0_ = there is no asymmetry) and indicating asymmetry in the data, consistent with the suspected publication bias from the funnel plots. Although asymmetry is detected in the Egger’s test for the overall values (z = 1.2310, *p* = 0.2183) this was not statistically significant. For the aggregated data the negative z value (z = −0.5939, *p* = 0.5526) supports the findings from the contour enhanced funnel plots that studies from the right-hand side of the funnel plot appear to be missing. 

Results of the rank test lead to the rejection of the null hypothesis, indicating that publication bias in present in all datasets. This was significant at the 0.1% level for the complete case (0.3103, *p* = 0.0001) and overall values (0.2594, *p* < 0.0001), and at the 5% level for the aggregated values (0.1944, *p* = 0.0392). The correlation was weakest for the aggregated dataset, which may be a reflection of the smaller number of values in this dataset, and also the studies missing from the right-hand side, as indicated from both the funnel plot and the Egger’s test.

The Vevea and Hedges test again confirmed that publication bias was present in the data, with there being statistically significant differences between the unadjusted and adjusted WTP estimates for the complete case χ_2_ (1) = 60.51, *p* < 0.0001, overall χ_2_ (1) = 81.28, *p* = 0.000) and aggregated values χ_2_ (1) = 10.03, *p* = 0.0015) as highlighted in Table 2. The significant results indicate the adjusted models fit the data better [50]. For both the complete case and the overall values, the adjusted values are lower than the initial estimates, with values becoming negative following adjustment, changing from 0.63 to −0.23 and from 0.57 to −0.25, respectively. It should be noted that this negative effect size does not imply that individuals are not willing-to-pay or wish to be compensated for purchasing higher-welfare products; rather, they expect them to have additional benefits, such as being healthier or safer products, or to have been produced in a more environmentally friendly manner [51]. For the aggregated data, the opposite was true, with the effect size increasing from 0.47 to 0.96, which is unsurprising as, from the previous analyses, it would appear that positive WTP estimates will have been added to create the adjusted estimate.

Finally, the mean effect size between published and unpublished studies was compared. There was little difference between them for all datasets (Table 1), with all having overlapping confidence intervals between published and unpublished values. For both complete case and aggregated values, peer-reviewed publications deliver slightly higher values, while for the overall dataset average published values are slightly lower, which is likely due to larger standard deviations obtained through imputation (as they were not reported).

Overall, it would seem that publication bias is suspected in this example of the WTP literature, and this was consistently demonstrated across all tests, particularly for the complete case values, compared to the other two datasets where values had been imputed. For the aggregated values, publication bias is suspected across all tests, barring the Egger’s test, although the slight negative correlation would appear to support the overall finding that smaller studies with larger effect sizes appear to be missing. Few studies appear to be missing from the left-hand side of the funnel plots, although all graphs were missing studies from areas of non-significance, implying that studies with non-significant results are not being published. The results of the subgroup analysis support these findings, with the Vevea and Hedges model indicating publication bias across all subgroups, and the contour-enhanced funnel plots, Egger, and rank tests indicating publication bias for the majority of the subgroups. Readers are directed to the supplementary data file for more information.

## 4. Discussion

The results of the analysis indicate asymmetry, and a likely relationship between effect size and study size in the data, which leads to the conclusion that publication bias is strongly suspected in studies of consumer behaviour, specifically within the field of WTP. It would appear that small studies, with non-significant or small WTP values, are not being published, leading to an overestimation of WTP, as when the detected bias is adjusted for using the Vevea and Hedges model, the mean effect size reduced considerably. The aggregated data indicated that small, and some large, studies with large WTP estimates are either not being published or are difficult to retrieve. For the smaller studies, this could be due to the publication policy with editors reluctant to publish studies with large WTP values based on a small sample size. For the larger studies, which were consistently missing across the different data values, individuals may not be willing, or able, to pay large amounts for welfare-friendly products, compared to conventionally-produced products. These reasons may also explain why there were few studies with non-significant results identified. 

As WTP can be used as a proxy for attitudes, these findings are supported by a qualitative systematic review on public attitudes to FAW which demonstrated that the public are concerned over FAW, primarily in relation to more modern, intensive production diseases breaching the concepts of humane treatment and naturalness that were central to good FAW [52] and animals living a good life. However, despite their concerns over FAW, the qualitative review identified a number of barriers to purchasing higher welfare products, and a number of dissonance strategies were adopted to enable the consumption of conventionally-produced products, without any feelings of guilt. The findings also provide some explanation of the adjusted WTP estimate provided by the Vevea and Hedges weight function model, indicating that the implications of a negative WTP and does not necessarily mean consumers are not willing to pay for higher-welfare products. Instead, this is likely to be attributed to consumers associating additional attributes other than the perceived better welfare for animals with higher-welfare products, such as product safety and quality [53,54], thereby expecting additional guarantees.

Results of the subgroup analysis presented in the supplementary information reflect findings from the overall publication bias assessment, indicating bias present within the results by region, animal type, population, and methodological factors. In addition, within the subgroup analysis some significant differences between variables were detected. However, due to the bias within the results, it is not clear whether this is due to suspected publication bias or if it is a true reflection of the data itself. Findings of the meta-analysis should, therefore, be interpreted with caution. 

The presence of publication bias in the results emphasizes the need for more formalised assessment procedures, to both detect it in the first instance and subsequently aid with the interpretation of the results, so as to ensure that confidence can be given in the recommendations and that transparency is provided regarding the strength of evidence presented. As WTP information is used in policy decision-making it is important that the potential for publication bias is recognised, tested for, and taken into consideration when conducting reviews of the economic valuation literature. This is important as systematic review and meta-analysis are being more frequently applied in the social sciences. As a consequence, there is a need to ensure that comprehensive search strategies are used, and appropriate tests for bias are conducted and interpreted correctly. As per other disciplines, ensuring consistency and transparency within best practice of the review and synthesis process is essential and guidance should be developed in light of this, especially when the evidence base is being put forward to inform policy decisions.

Awareness of the problem of publication bias is a necessary pre-requisite for change [55] and although unpublished work can be tracked using all reasonable measures available [49], this can be very time consuming and may not be feasible. In other disciplines, increased use of meta-analysis has caused a re-evaluation of publication practices [48] so as to avoid the problem of publication bias in the first instance by eliminating differential selection [45]. The most common method of attempting to avoid publication bias is to encourage reporting mechanisms, such as trial registration (as per medicine and other social science disciplines) [13] to enable a complete database of studies to be built up [21]. However, few are currently available in the social sciences [22], and those that do exist are very domain-specific. The only long-term solution to the problem would, therefore, appear to be a change in scientific publication practices [20], both to ensure guidelines for primary research are published and to enable preventative measures to be put into place. The former will provide a means of ensuring that enough information is provided to enable meta-analysis and corresponding sensitivity analysis, whilst the latter are required to prevent publication bias from occurring in the first place [13]. 

Organisations, such as the Centre for Open Science [56], encourage the preregistration of research and data availability to increase inclusivity and transparency in research [57]. The increased use of supplementary data that facilitates this, and the use of best practices, should be encouraged, along with considerations, such as making original datasets available for access by other researchers and the consideration on limits on the size and number of files allowed [11]. Additionally, it is worth the consideration and encouragement of publication in lower-tier journals [57], including from the removal of a focus from publishing in top-tier journals only by various sources, so as to increase the chances of research being made publically available. Efforts from multiple stakeholders to achieve the successful implementation and adoption of such systems to ensure their completeness, comprehensiveness, and accessibility is needed [10,55], and it will take a comprehensive effort to achieve this. 

A number of potential limitations of this research should be considered. Firstly, problems with the heterogeneity of the data may affect the interpretation of the results due to moderating effects [11]. Heterogeneity is likely to be common within this field and it should be acknowledged that it makes the detection of publication bias more complex, especially for the Egger and Rank tests. However, despite the heterogeneity present, it is important to note that, in some instances, it is still more useful to look at the effects across studies rather than looking at the results of one study individually. The only way to get around this issue would be to look at the raw data from each identified study, and this can be difficult and time consuming to obtain, and may not be possible in all instances, Finally, although all meta-analyses may not be influenced by publication bias [23], it is still important to test for this to provide an indication as to the strength of the results as part of a sensitivity analysis, and this is simply not currently being done. 

In addition, there is always the need to consider that the publication bias may not be due to statistical significance. It could be due to any factor that influences both the study effect and the study size [58], such as the large number of studies with small effect sizes and the greater flexibility in research designs and analytical methods available to assess WTP [59]. In order to establish the cause of this apparent bias, the prevalence and extent of publication bias in reviews of other WTP studies should be assessed. However, as the different methods used to assess publication bias arrive at the same conclusion, greater confidence can be had that publication bias is likely present in the data [11].

## 5. Conclusions

This study sought to explore publication bias in the WTP literature, using public WTP for FAW as an example. Results indicate that publication bias is present in the literature, both overall and at the subgroup level. When adjusted for, the bias would appear to make the study effect disappear. Future systematic reviews and meta-analyses within the field should, therefore, look to test for publication bias as a form of sensitivity analysis as standard, and guidance should be developed as to the best practices for this. Procedures and practices should also be developed to try to prevent publication bias in the first instance, by facilitating the registry and publication of studies. 

## Figures and Tables

**Table 1:** Results from five assessments of publication bias.

Test		Data Values		
		Complete Case	Aggregated	Overall
**Egger test (z score)**		3.7300, *p* = 0.0002 ***	−0.5939, *p* = 0.5526	1.2310, *p* = 0.2183
**Ranktest (Kendalls tau)**		0.3103, *p* = 0.0001 ***	0.1944, *p* = 0.0392 *	0.2594, *p* < 0.0001 ***
**Contour enhanced funnel plot**		Yes	Yes	Yes
**Average effect size (95% CI)**	**Peer reviewed**	0.6390 (0.4858, 0.7922)	0.4798 (0.1861, 0.7736)	0.5654 (0.4393, 0.6914)
	**Non-peer reviewed**	0.5839 (0.4393, 0.6914)	0.4003 (−0.0666, 0.8671)	0.6017 (0.4367, 0.7666)
**Number measures**		227	54	335
**Number studies**		37	54	54
**Model results (effect size, 95% CI, I^2^)**		0.6302 (0.5016, 0.7587)	0.6135 (0.4106, 0.8524)	0.5709, (0.4599, 0.6819)

Significant at: * 0.05, *** 0.001.

**Table 2:** Results from the Vevea and Hedges assessment of publication bias at the 0.05 cutpoint.

Model Component	Data Values		
	Case Complete	Aggregated	Overall
*n*	227	54	335
Unadjusted model intercept	0.63 ± 0.07	0.47 ± 0.13	0.57 ± 0.06
Unadjusted model variance component	0.97 ± 0.09	0.94 ± 0.18	1.06 ± 0.08
Adjusted model intercept	−0.23 ± 0.18	0.96 ± 0.18	−0.25 ± 0.14
Adjusted model variance component	1.40 ± 0.16	0.76 ± 0.13	1.44 ± 0.13
df	1	1	1
2*difference	60.51	10.03	81.28
Likelihood ratio test	*p* < 0.05	*p* < 0.05	*p* < 0.05

Results presented with standard error values.

## Supplementary Materials

The following are available online at www.mdpi.com/2076-2615/7/3/23/s1. Table S1: Keywords included in the search; Table S2: Results of the subgroup analysis; Figure S1: Cumulative forest plot by date of publication; Supplementary information S1: Studies included in the review.
