# Supplementary material for: The Need for Formal Evidence Synthesis in Food Policy: A Case Study of Willingness-to-Pay
Source: Animals (Basel). 2017 Mar 10;7(3):23. doi: 10.3390/ani7030023 (PMC5366842; doi:10.3390/ani7030023)
Supplement: Supplementary file 1 [file animals-07-00023-s001.pdf]

The Need for Formal Evidence Synthesis in Food Policy: a Case Study of Willingness-to-Pay

Beth Clark[1], Lynn J. Frewer[1], Luca A. Panzone[1], Gavin B Stewart[1*]

[1]School of Agriculture Food and Rural Development, Newcastle University, Newcastle upon Tyne, NE1 7RU, United Kingdom

*Corresponding Author

Email: gavin.stewart@newcastle.ac.uk

Supplementary Table S1: Keywords Included in the Search

**Type of Study and Outcome**	valu* OR intention* OR behav* OR purchas* OR WTP OR willingness to pay OR willingness to buy OR ITP OR buy OR pref*OR economic OR reject* OR consumer OR demand OR choice
**AND**	
**Animal Type**	farm animal OR production animal
**AND**	
**Animal Welfare**	animal welfare OR health OR disease OR welfare OR production disease

Supplementary Figure S1: Cumulative Forest Plot by Date of Publication

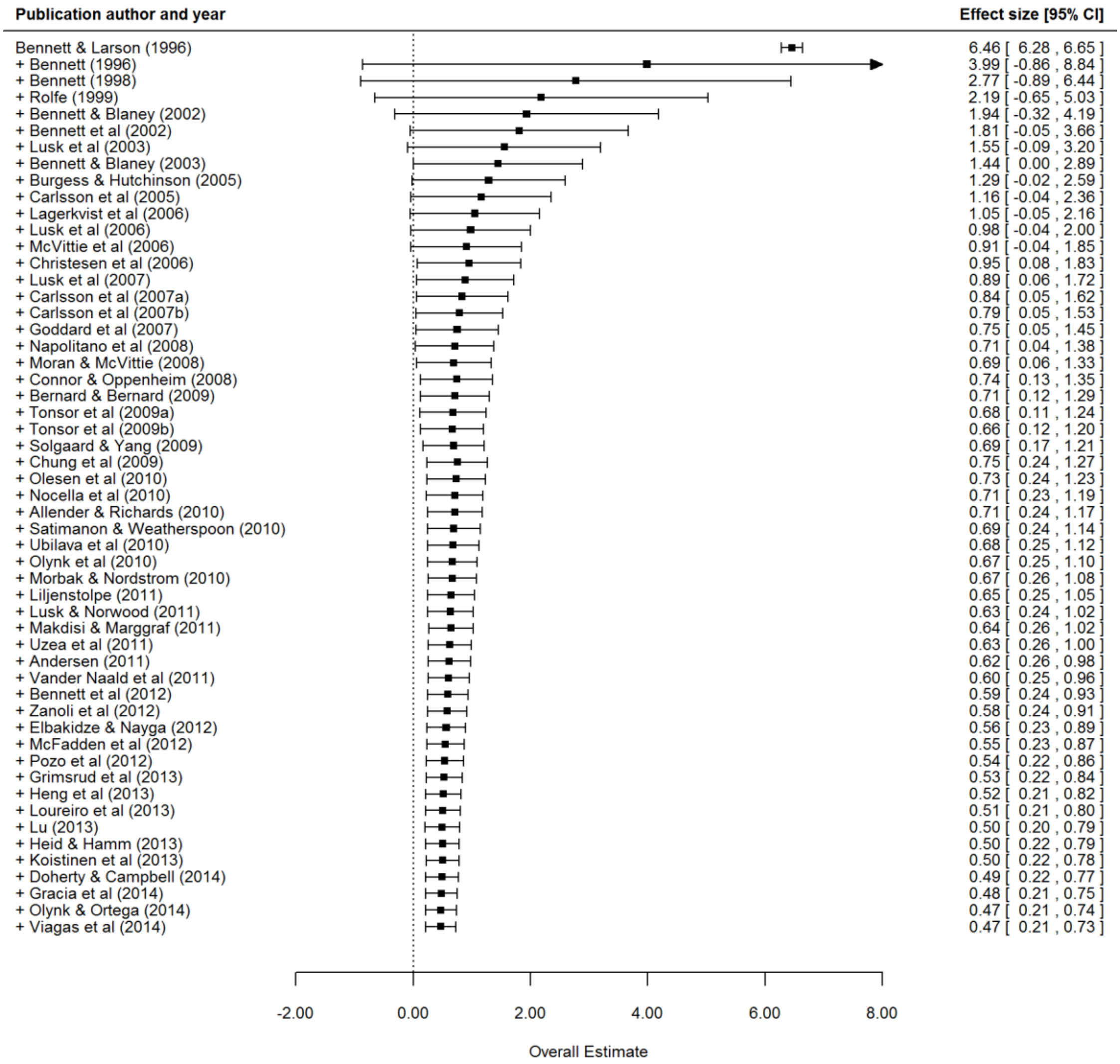

Supplementary Information S1: Studies Included in the Review

- Allender, W.J. & Richards, T.J. (2010). Consumer Impact of Animal Welfare Regulation in the California Poultry Industry, *Journal of Agricultural and Resource Economics*, 35(3), pp. 424-442.

- Andersen, L.M. (2011). Animal Welfare and Eggs - Cheap Talk or Money on the Counter? *Journal of Agricultural Economics*, 62(3), pp. 565-584.

- Bennett, R. (1998). *Measuring public support for animal welfare legislation: A case study of cage egg production,* Animal Welfare, 7(1), pp. 1-10.

- Bennett, R. & Blaney, R. (2002). Social consensus, moral intensity and willingness to pay to address a farm animal welfare issue, *Journal of Economic Psychology*, 23(4), pp. 501-520.

- Bennett, R., Kehlbacher, A. & Balcombe, K. (2012). *A method for the economic valuation of animal welfare benefits using a single welfare score,* Animal Welfare, 21, pp. 125-130.

- Bennett, R. & Larson, D. (1996) Contingent valuation of the perceived benefits of farm animal welfare legislation: an exploratory survey, *Journal of Agricultural Economics*, 47(1-4), pp. 224-235.

- Bennett, R.M. (1996) People's willingness to pay for farm animal welfare, *Animal Welfare*, 5(1), pp. 3-11.

- Bennett, R.M., Anderson, J. & Blaney, R.J.P. (2002). Moral intensity and willingness to pay concerning farm animal welfare issues and the implications for agricultural policy, *Journal of Agricultural & Environmental Ethics*, 15(2), pp. 187-202.

- Bennett, R.M. & Blaney, R.J.P. (2003). Estimating the benefits of farm animal welfare legislation using the contingent valuation method, *Agricultural Economics*, 29(1), pp. 85-98.

- Bernard, J.C. & Bernard, D.J. (2009).What is it about organic milk? An experimental analysis, *American Journal of Agricultural Economics*, 91(3), pp. 826-836.

- Burgess, D., Hutchinson, W.G., McCallion, T. and Scarpa, R. (2003). Investigating choice rationality in stated preference methods for enhanced farm animal welfare, *Working Paper - Centre for Social and Economic Research on the Global Environment*. pp. 1-36. Available at: http://www.scopus.com/inward/record.url?eid=2-s2.0-55849094325&partnerID=40&md5=5a49c0ff3c953c806ff1b40c5c60935f.

- Carlsson, F., Frykblom, P. & Lagerkvist, C.J. (2005). Consumer preferences for food product quality attributes from Swedish agriculture, *AMBIO: A Journal of the Human Environment*, 34(4), pp. 366-370.

- Carlsson, F., Frykblom, P. & Lagerkvist, C.J. (2007a). Consumer willingness to pay for farm animal welfare: mobile abattoirs versus transportation to slaughter, *European Review of Agricultural Economics*, 34(3), pp. 321-344.

- Carlsson, F., Frykblom, P. & Lagerkvist, C.J. (2007b). Farm animal welfare-testing for market failure, *Journal of Agricultural and Applied Economics*, 39(1), p. 61.

- Christensen, T., Mørkbak, M., Hasler, B., Lundhede, T., Porsbo, L.J. & Christoffersen, L.B. (2006). Information, risk perception and consumer behaviour: a choice experiment on food safety and animal welfare. Copenhagen. Fødevareøkonomisk Institut. (Rapport / Fødevareøkonomisk Institut; No. 180).

- Chung, C., Boyer, T. & Han, S. (2009). Valuing quality attributes and country of origin in the Korean beef market, *Journal of Agricultural Economics*, 60(3), pp. 682-698.

- Conner, D.S. & Oppenheim, D. (2008). Demand for pasture-raised livestock products: results from Michigan retail surveys, *Journal of Agribusiness*, 26(1), pp. 1-20.

- Doherty, E. & Campbell, D. (2014). Demand for safety and regional certification of food Results from Great Britain and the Republic of Ireland, *British Food Journal*, 116(4), pp. 676-689.

- Elbakidze, L. & Nayga, R.M., Jr. (2012). The effects of information on willingness to pay for animal welfare in dairy production: Application of nonhypothetical valuation mechanisms, *Journal of Dairy Science*, 95(3), pp. 1099-1107.

- Goddard, E.W., Boxall, P., Emunu, J.P., Boyd, C., Asselin, A. & Neall, A. (2007). *Consumer attitudes, willingness to pay and revealed preferences for different egg production attributes: analysis of Canadian egg consumers*. Department of Rural Economy, University of Alberta.

- Gracia, A., Barreiro-Hurle, J. & Lopez-Galan, B. (2014). Are Local and Organic Claims Complements or Substitutes? A Consumer Preferences Study for Eggs, *Journal of Agricultural Economics*, 65(1), pp. 49-67.

- Grimsrud, K.M., Nielsen, H.M., Navrud, S. & Olesen, I. (2013). Households' willingness-to-pay for improved fish welfare in breeding programs for farmed Atlantic salmon, *Aquaculture,* 372, pp. 19-27.

- Heid, A. & Hamm, U. (2013). Animal welfare versus food quality: Factors influencing organic consumers' preferences for alternatives to piglet castration without anaesthesia, *Meat Science*, 95(2), pp. 203-211.

- Heng, Y., Peterson, H.H. & Li, X. (2013). Consumer attitudes toward farm-animal welfare: The case of laying hens, *Journal of Agricultural and Resource Economics*, 38(3), pp. 418-434.

- Koistinen, L., Pouta, E., Heikkilä, J., Forsman-Hugg, S., Kotro, J., Mäkelä, J. & Niva, M. (2013). The impact of fat content, production methods and carbon footprint information on consumer preferences for minced meat, *Food Quality and Preference,* 29(2), pp. 126-136.

- Lagerkvist, C.J., Carlsson, F. & Viske, D. (2006). Swedish consumer preferences for animal welfare and biotech: a choice experiment. *The Journal of Agrobiotechnology Management & Economics,* 9(1).

- Liljenstolpe, C. (2011). Demand for Value-Added Pork in Sweden: A Latent Class Model Approach, *Agribusiness*, 27(2), pp. 129-146.

- Loureiro, M.L., Gracia, A. & Nayga, R.M., Jr. (2013). Do experimental auction estimates pass the scope test?, *Journal of Economic Psychology*, 37, pp. 7-17.

- Lu, Y. (2013). *Consumer Preference for Eggs from Enhanced Animal Welfare Production System: A Stated Choice Analysis.* PhD Thesis, University of Guelph.

- Lusk, J.L., Nilsson, T. & Foster, K. (2007). Public preferences and private choices: effect of altruism and free riding on demand for environmentally certified pork, *Environmental and Resource Economics*, 36(4), pp. 499-521.

- Lusk, J.L. & Norwood, F.B. (2012). Speciesism, altruism and the economics of animal welfare, *European Review of Agricultural Economics*, 39(2), pp. 189-212.

- Lusk, J.L., Norwood, F.B. & Pruitt, J.R. (2006). Consumer demand for a ban on antibiotic drug use in pork production, *American Journal of Agricultural Economics*, 88(4), pp. 1015-1033.

- Lusk, J.L., Roosen, J. & Fox, J.A. (2003). Demand for beef from cattle administered growth hormones or fed genetically modified corn: a comparison of consumers in France, Germany, the United Kingdom, and the United States, *American Journal of Agricultural Economics*, 85(1), pp. 16-29.

- Makdisi, F. & Marggraf, R. (2011). *Consumer willingness to pay for farm animal welfare in Germany – the case of broiler chickens. 30th* September, Halle, Germany. Proceedings of the 51st Annual Conference.

- McFadden, B.R., Lusk, J.L., Crespi, J.M., Cherry, J.B.C., Martin, L.E. & Bruce, A.S. (2012). *Effect of advocacy information on consumer preferences for cage free eggs: a neuroeconomic analysis.* 12-14th August, Seattle, Washington. Agricultural and Applied Economics Association Annual Meeting.

- McVittie, A., Moran, D. & Nevison, I. (2006). Public preferences for broiler chicken welfare: evidence from stated preference studies, Land Economy Paper Series, Land Economy Research Group, Scottish Agricultural College. Available at: http://www.sac.ac.uk/research/publications/lee/workingpapers.

- Moran, D. & McVittie, A. (2008). Estimation of the value the public places on regulations to improve broiler welfare, *Animal Welfare*, 17(1), pp. 43-52.

- Morten Raun, M., Tove, C. & Dorte, G.H. (2010). Consumer preferences for safety characteristics in pork, *British Food Journal*, 112(7), pp. 775-791.

- Napolitano, F., Pacelli, C., Girolami, A. & Braghieri, A. (2008). Effect of information about animal welfare on consumer willingness to pay for yogurt, *Journal of Dairy Science*, 91(3), pp. 910-917.

- Nocella, G., Hubbard, L. & Scarpa, R. (2010). Farm animal welfare, consumer willingness to pay, and trust: Results of a cross-national survey, *Applied Economic Perspectives and Policy,* 32(2), pp. 275-297.

- Olesen, I., Alfnes, F., Rora, M.B. & Kolstad, K. (2010). Eliciting consumers' willingness to pay for organic and welfare-labelled salmon in a non-hypothetical choice experiment, *Livestock Science*, 127(2-3), pp. 218-226.

- Olynk, N.J., Tonsor, G.T. & Wolf, C.A. (2010). Consumer Willingness to Pay for Livestock Credence Attribute Claim Verification, *Journal of Agricultural and Resource Economics*, 35(2), pp. 261-280.

- Pozo, V.F., Tonsor, G.T. & Schroeder, T.C. (2012). How Choice Experiment Design Affects Estimated Valuation of Use of Gestation Crates, *Journal of Agricultural Economics*, 63(3), pp. 639-655.

- Rolfe, J. (1999). Ethical rules and the demand for free range eggs, *Economic Analysis and Policy*, 29(2), pp. 187-206.
- Satimanon, T. & Weatherspoon, D.D. (2010). Hedonic Analysis of Sustainable Food Products, *International Food and Agribusiness Management Review*, 13(4), pp. 57-74.
- Solgaard, H.S. & Yang, Y. (2011). Consumers' perception of farmed fish and willingness to pay for fish welfare, *British Food Journal*, 113(8-9), pp. 997-1010.
- Tonsor, G.T., Olynk, N. & Wolf, C. (2009a). Consumer preferences for animal welfare attributes: The case of gestation crates, *Journal of Agricultural and Applied Economics*, 41(3), pp. 713-730.
- Tonsor, G.T., Wolf, C. & Olynk, N. (2009b). Consumer voting and demand behavior regarding swine gestation crates, *Food Policy*, 34(6), pp. 492-498.
- Ubilava, D., Foster, K.A., Lusk, J.L. & Nilsson, T. (2010). Effects of income and social awareness on consumer WTP for social product attributes, *Technological Forecasting and Social Change*, 77(4), pp. 587-593.
- Uzea, A.D., Hobbs, J.E. & Zhang, J. (2011). Activists and Animal Welfare: Quality Verifications in the Canadian Pork Sector, *Journal of Agricultural Economics*, 62(2), pp. 281-304.
- Vander Naald, B. & Cameron, T.A. (2011). Willingness to pay for other species' well-being', *Ecological Economics*, 70(7), pp. 1325-1335.
- Viegas, I., Nunes, L.C., Madureira, L., Fontes, M.A. & Santos, J.L. (2014). Beef Credence Attributes: Implications of Substitution Effects on Consumers' WTP', *Journal of Agricultural Economics*, 65(3), pp. 600-615.
- Widmar, O., Nicole, J., Ortega, D.L., McKenzie, A. & Paudel, K. (2014). Comparing Consumer Preferences for Livestock Production Process Attributes Across Products, Species, and Modeling Methods, *Journal of Agricultural and Applied Economics*, 46(03).
- Zanoli, R., Scarpa, R., Napolitano, F., Piasentier, E., Naspetti, S. & Bruschi, V. (2013). Organic label as an identifier of environmentally related quality: A consumer choice experiment on beef in Italy, *Renewable Agriculture and Food Systems*, 28(1), pp. 70-79.

Supplementary Table S2: Results of the Subgroup Analysis.

	No. measures	No. studies	I2	Effect size (95% CIl)	Regtest (z value)	Ranktest (Kendalls Tau)	Funnel plot	Vevea & Hedges model
**Animal type**[1]								
Beef cow	24	7	99.84	1.2022 (0.7294, 1.6750)	0.7436, p = 0.4571	0.1888, p = 0.2106	Yes	p < 0.001***
Pig	90	13	98.33	0.2843 (0.1936, 0.3750)	2.4579, p = 0.014**	0.1680, p = 0.0239**	Yes	
Layer hen	47	10	99.88	0.7823 (0.3594, 1.2053)	1.1088, p = 0.2675	0.2551, p = 0.0163**	Yes	
Broiler chicken	26	8	97.92	0.4024 (0.2653, 0.5394)	4.1308, p < 0.0001***	0.5364, p = 0.0002***	Yes	
Mixed	6	2	94.78	0.6547 (0.4206, 0.8888)	–0.0606, p = 0.9516	0.0667, p = 1.0000	Yes	
Dairy cow	27	7	99.53	1.1176 (0.7776, 1.4575)	2.8086, p–0.005***	0.3395, p = 0.0177	Yes	
Fish	6	3	99.29	0.3712 (–0.0073, 0.7497)	–0.4668, p = 0.6406	–0.0861, p = 0.8216	Yes	
**Population**								
Citizens	187	26	99.65	0.5122 (0.3810, 0.6435)	3.7755, p = 0.0002***	0.2845, p < 0.0001***	Yes	p < 0.001***
Consumers	40	11	99.79	1.1796 (0.8287, 1.5304)	1.6097, p = 0.1075	0.1634, p = 0.1545	Yes	
**Region**								
UK	27	7	97.07	0.6479 (0.5113, 0.7845)	1.9722, p = 0.0486**	0.2357, p = 0.3920	Yes	p < 0.001***
Northern Europe	76	8	97.84	0.1060 (0.0376, 0.1744)	–2.8201, p = 0.0048***	–0.1551, p = 0.0655	No	
Western Europe	7	3	94.66	1.0741 (0.7720, 1.3763)	2.0213, p = 0.4320***	0.3504, p = 0.2820	Yes	
North America	90	16	99.74	0.7515 (0.5026, 1.0004)	1.0488, p = 0.2943	0.2296, p = 0.0019**	Yes	
Southern Europe	23	6	99.73	1.4329 (0.9577, 1.9082)	2.3457, p = 0.0190**	0.2967, p = 0.0596*	Yes	
**Use of a cheap talk script**								
Script used	83	9	99.39	0.3595 (0.2259, 0.4932)	1.4715, p = 0.1412	0.2359, p = 0.0027**	No	p < 0.001***
Script not used	113	20	99.79	0.6758 (0.4600, 0.8916)	1.9413, p = 0.0522	0.2083, p = 0.0015**	Yes	
Script not needed	31	8	98.72	1.1935 (0.9077, 1.4793)	0.3713, p = 0.7104	0.0334, p = 0.7974	No	
**Willingness to pay method**								
Revealed preference	31	8	98.72	1.1935 (0.9077, 1.4793)	0.3713, p = 0.7104	0.0334, p = 0.7974	No	p < 0.001***
Stated preference	196	29	99.73	0.5416 (0.4035, 0.6796)	2.7402, p = 0.0061**	0.2563, p < 0.0001***	Yes	

[1] Calves and Asia not included in the analysis due to their only being one study for each respective subgroup analysis
